# Exploring synergies and performance evaluation between Islamic funds and socially responsible investment (SRIs) in light of the Sustainable Development Goals (SDGs)

**DOI:** 10.1016/j.heliyon.2020.e04562

**Published:** 2020-08-22

**Authors:** Abdurahman J. Yesuf, Dalal Aassouli

**Affiliations:** a College of Islamic Studies, Hamad Bin Khalifa University, Education City, Al-Rayyan, Qatar; b Assistant Professor of Islamic Finance, College of Islamic Studies, Hamad Bin Khalifa University, Education City, Al-Rayyan, Qatar

**Keywords:** Islamic finance, Islamic funds, Socially responsible investments, Islamic sustainable investing fund, Financial market, Willingness-to-Pay, Corporate social responsibility, Business

## Abstract

This study aims to evaluate risk and return characteristics of Islamic funds in comparison with SRI and investigate the possible synergies between the two in the light of the SDGs. The study also compares the financial performance of Islamic funds with conventional funds and Islamic market benchmarks. The analysis was carried out using the absolute and risk-adjusted-performance evaluation techniques based on data collected from 11 countries distributed in four geographical regions. The results demonstrate that there was no statistically significant difference between the returns of Islamic funds and SRI funds in all regions. Islamic funds were also the least risk-sensitive instruments compared to their counterparts in most of the regions. The results indicate that embedding ESG/SDGs considerations into Islamic funds investment decisions do not adversely affect their returns. Rather, it enhances their positive impact and contribution to mitigate the SDGs financing gaps. The analysis further demonstrates the possible synergies between the two categories of funds in line with *Shariah* principles. Hence, the study highlights the importance of developing a new asset class, “*Shariah*-compliant SRIs”, that is both *Shariah*-compliant and integrates ESG/SDGs considerations. The new asset class will target a wider investor base including both *Shariah* and impact investors, which will support the achievement of the SDG agenda.

## Teaser

Islamic finance, Islamic funds, Sustainable investing, Socially responsible investments, Islamic sustainable investing fund; Financial market; Willingness-to-Pay; Sustainable business; Corporate governance; Corporate social responsibility; Business.

## 1 Introduction

A real economy serving the society needs financial institutions, individuals, and enterprises that prioritize societal value over their profit-maximizing goals. Their investments should be directed into activities that produce not only economic resilience but also environmental regeneration and social empowerment for the communities and people they serve (GABV, 2017). In 2015, global leaders endorsed the 17 Sustainable Development Goals (SDGs), thus providing a framework to identify and fund socially beneficial investment. Achieving these aspiring goals; however, requires significant investments. According to UNCTAD, achieving the SDGs requires an annual investment between $5 trillion to $7 trillion with an investment gap of about $2.5 trillion in developing countries (Niculescu, 2017). This gap cannot be mitigated by governments alone and requires the private sector's active participation.

Although in today's world, the financial system is in different ways too large and too complex, it mainly focusses on short-term profit maximization without paying enough attention to social and environmental risks (UNEP, 2017). On the other hand, there are growing concerns about the moral aspects of doing business among investors and corporations. Big companies are facing pressures to act in a responsible way towards multiple sets of stakeholders, consumers demand increasing transparency in business practices, and environmentally or socially-minded innovators excite investors and consumers alike about a more sustainable future (GIIN, 2018). In this regard, the United Nations (UN) has launched several principles in 2006 to promote and guide responsible investing. Even though these principles suggest voluntary compliance, they officially recognize the importance of embedding ESG objectives in the financial sector, thus creating awareness about impact and sustainability issues. As a result, several institutional investors have already accepted the UN principles and incorporated ESG matters into their investment decisions.

Sustainable or impact investments, where positive impacts are sought alongside financial returns, play a significant role in providing funds and technical assistance to contribute to the achievement of the SDGs. Among many other investments, ‘ethics’ based (the so-called Socially Responsible Investing or SRI) and faith-based investments (also called Morally Responsible Investment (MRI) funds such as Islamic funds) represent the two growing segments of impact investing Globally (Ghoul and Karam, 2007). The two investments promote the achievement of the SDGs as they take into consideration the well-being of the society in their investment strategies while providing reasonable return to investors. Due to this, many observers stressed the growing importance and acceptance of mainly SRIs by several funds and made the concept unavoidable (Luc et al., 2008).

SRIs or impact investing refer to funds that emphasize social, financial, and environmental attributes in the process of selecting an investment portfolio (Camilieri, 2017). They are defined in different ways but, in general, they refer to investment strategies that consider environmental, social, and governance (ESG) factors in investment decision making, portfolio selection, and management process (Chatzitheodorou et al., 2019). Sustainable investments, where positive impacts are sought alongside financial returns, play a significant role in providing funds and technical assistance to mitigate the SDG financing gaps. During the past few years, SRIs have demonstrated their potential in mitigating the SDGs financing gap especially in the EU and North America (IRTI, 2017). With regard to their geographical distributions, more than 90% of the SRIs are also domiciled in Europe and North America (GSIA, 2018).

On the other hand, faith based or MRI funds (such as Islamic funds) represent a very small portion of the sustainable investment universe. Unlike SRIs which witnessed significant growth globally during the past two decades, the universe of Islamic investment funds is still small in size and relatively new compared to SRIs. The size of the Islamic fund segment represents only $108 billion or 4% of the Islamic finance industry's total assets in 2018 whereas more than 70% of the Islamic finance industry's assets are concentrated in the banking sector (Refinitiv, 2019). Although the majority of Islamic funds are found in Muslim or OIC member countries, their investments are not sufficient to finance SDGs in the region. There are huge investment needs in the region to mitigate the existing SDGs financing gaps (IRTI, 2017). In these countries, SRI assets which provide sustainable financing for the SGDs are very limited. On the other hand, the majority of Muslim countries that are Islamic finance hubs, confront several development challenges that harm the well-being of the society^1^. Therefore, it is critical to develop synergies between SRIs and MRIs, especially Islamic funds, in order to mobilize additional investment to support the achievements of the SDGs.

Since both Islamic funds and SRIs have shared objectives and similar characteristics (Chowdhury and Masih, 2015), it is also interesting to assess whether they have any differences in their financial performance. Evaluating the risk-return characteristics of SDG-driven Islamic funds in comparison with SRI is important to understand the effect of integrating ESG/SDG considerations into their investment decisions. Hence, the first objective of the study is to make a comparison between Islamic funds that have a direct contribution to SDGs and their conventional counterparts, mainly SRIs. The intention is to demonstrate the risk and return characteristics of ‘SDG-oriented Islamic funds’ in comparison primarily with the SRIs. The second objective of the study is to explore the possible synergies between Islamic funds and SRIs. The purpose is to assess the possibility of introducing “*Shariah*-compliant SRIs” that are both *Shariah*-compliant and integrates ESG/SDG and SRI considerations. The overall aim is to promote the achievement of the SDGs especially in Muslim countries where SRI assets are very limited.

Therefore, in the first part of the analysis, we investigate the risk-adjusted return characteristics and riskiness of Islamic funds in comparison with the SRIs as well as the conventional funds and Islamic market with the aim of enlightening both Islamic and conventional investors. The second part of the analysis explores the potential synergies between Islamic funds and SRIs in line with *Shariah* principles and suggest the creation of a new asset class targeting a wide investor base of ethical and SRI investors.

## 2 Related literature and hypotheses

### 2.1 Socially responsible investments (SRIs)

Globally, there are different terminologies used to describe SRIs. These include sustainable investing, ‘ethical’ investing or fund, impact investing, green investing, and responsible investing (Ghoul and Karam, 2007). The lack of standard definition is also seen in the existing literature on SRIs. Among others, Weigand et al. (1996) defined SRIs as “a type of investment that takes into account ethical and social considerations, in addition to the traditional financial objectives in the selection of the securities integrating an investment portfolio”. The Global Sustainable Investment Review described ‘sustainable investing’ as an investment approach that considers ESG factors in the process of portfolio formation and management (GSIA, 2016). It has also been explained by Lowry (1993) as a screening process of identifying and investing in business or activities that can create a positive impact on society and the environment while generating economic profits. However, there is a common understanding in most of the definitions that SRIs are investment alternatives that give priority to earning maximum profits while taking into account ESG parameters and pursuing a positive impact. They take into account both investment's impact on society and investors' financial needs (returns). Hence, investors are obliged to make a choice of value and company or projects reflecting their beliefs and desire for changes in the company (Argandona and Sarsa, 2000).

The rising interest of investors and the positive contribution of SRIs to SDGs impels many researchers to explore investors' motivations to invest in SRIs. There are different factors that compel impact investors to make their investments in a sustainable way. Among many others researchers, Rosen et al. (1991), Anand and Cowton (1993), O'Neil and Pienta (1994), Beal and Goyen (1998), Lewis and Mackenzie (2000), McLachlan and Gardner (2004), Brammer and Pavelin (2006), and Haigh (2007) discussed the different motives of socially responsible investors. In the 1970s, the motive of socially responsible investors was to create equal opportunities for all citizens through their investments. Malkiel and Quandt (1971) indicated that one of the motives of modern sustainable investing was to ensure equal opportunities for everyone. They argue that to attain the objectives of the SRIs, all stakeholders participating in investment decision making should take into consideration the moral consequences, political impact, and social impact of their investments. Investors were also motivated to use their action to try to change business practices that are contrary to the so-called social responsibility and to exclude ‘socially irresponsible’ companies from their investing lists (Cowton, 1992).

Heard (1978) also argues that ‘ethical’ or socially responsible investors act according to the assumed or expected moral obligation of organizations which is not to cause harm to society. Investors invest rationally to encourage activities and organizations that produce positive externalities through involving in it and discouraging those activities that have negative externalities through exclusion or rejecting investments in these activities (Rudd, 1981). Traditionally, socially responsible investing is also linked to the so-called ‘clean products’ and avoid ‘Sin activities’ including alcohol production and distribution, tobacco production, investing in the pornography industry, gambling, and involving in mass destruction weapons (Bruyn, 1987).

Investors follow different SRIs strategies and guidelines to achieve their sustainable investment goals. There are various SRIs guideline setting institutions; however, the Social Investment Forum (SIF) is the most influential and popular in guidelines setting for SRIs (Ghoul and Karam, 2007). Among others, the most widely used strategy is the screening strategy. This investment strategy involves verifying whether companies consider certain ESGs, ethical, and good corporate governance criteria while conducting their business. The European SRIs Study group (EUROSIF, 2018) and GSIA (2016 &^2018^) identify seven basic categories of SRIs strategies. They are Sustainability-themed investment; Best-in-Class investment selection; Exclusion from investment universe; Norms-based screening; ESG Integration factors; Engagement and voting on issues related to sustainability; and Impact based investing.

Similarly, the UK based SRIs Service (2019) categorizes the different strategies adopted by sustainable investors or SRIs funds managers into three categories. These are positive screening approach which aims at supporting companies that engage in impactful activities and beneficial things to the society and the environment (positive screening); negative screening approach or excluding business organizations that engage in activities that the fund has committed to exclude or have negative externalities (negative screening); and engaging for positive change (integrating SDGs and ESGs) approach.

### 2.2 Islamic finance and investment funds

Islamic finance refers to a financial system that operates in accordance with *Shariah* principles and Islamic teachings. Unlike the conventional system, Islamic finance's implementation should fall in with the main tenets of *Shariah,* thus, considered as a *Shariah*-compliant financial system (Gait and Worthington, 2008). This means that investing or financing certain types of goods and activities that do not comply with Islamic law is prohibited. Thus, it is not permitted to make investments towards economic entities or sectors that engage in activities considered as unlawful (*Haram*) in Islam which include producing alcohol, tobacco, and harmful goods, doing business in gambling industries, investing in the pornography industry, smuggling, pork and business entities with high leverage (Farid, 2008).

Such exclusion principle in Islamic finance is one of the similarities between *Shariah*-compliant investments and SRI's as the latter use negative screening strategies. In Islamic finance, investors use the *Shariah* screening criteria as a guiding principle to ensure that the sectors and business activities they invest in are not against any *Shariah* principles (Khatkhatay and Nisar, 2006). The screening criteria used in filtering Islamic investments are two types: Qualitative and Quantitative Screening. The qualitative screening is also known as business screening. As per this screening, all businesses involved in activities which contradict with the Islamic *Shariah* principles are excluded from the investable universe. The quantitative, also known as financial, screening techniques are used to exclude companies with a significant portion of their assets is financed by debt (Yildirim and Ilham, 2018).

The Islamic finance industry has been growing rapidly and its popularity increasing during the last four decades. In 2018, more than 61 countries reported the existence and services of different Islamic financial institutions and the total asset values of the Islamic finance industry amounted to US$ 2.52 trillion (Refinitiv, 2019).

The Islamic finance industry divided into five key segments or sectors. These are the Banking sector, Islamic insurance (Takaful), Other Islamic Financial Institutions, Islamic capital market or Sukuk, and Islamic Funds. According to the Islamic finance development report 2018, the banking sector is the largest sector of the Islamic finance industry with about 71% of the total assets of the industry, estimated to be $1.721 Trillion in 2017. The *Sukuk* market, the second largest segment, represents 17% of the industry's total assets and amounted to $426 billion of Sukuk outstanding in 2017. The Other Islamic Financial Institutions (which include microfinance institutions and investment companies with total assets of $135 billion), the Takaful sub-sector (total assets of $46 Billion) and the Islamic Funds (asset management) sub-sectors ($110 billion) take the third, fourth and fifth positions respectively based on their total asset values. After all, the financial institutions (sub-sectors, i.e. banks, *takaful*, etc.) are considered as the backbone of the industry while capital market asset classes (i.e. Sukuk issuance and Islamic funds or Asset under Management) play a significant role as investment instruments (Thomson Reuters, 2018).

The Islamic fund sector represents the fourth largest share of the Islamic finance industry assets (Refinitiv, 2019). Islamic funds differ from conventional funds in their management process which is based on ethical principles that are derived from Islamic law. According to Islamic principles, investments should be free from basic prohibition including *Riba*^2^ (interest or usury), *Gharar*^3^, *Maysir*^4^, and other prohibitions. However, both funds have common features and shared objectives, such as pooling investors' money, preserving the capital, and optimizing financial returns.

The Accounting and Auditing Organization for Islamic Financial Institutions (AAOFI) defines funds as follows^5^:

“*Funds are investment vehicles, which are financially independent of the institutions that established them. Funds take the form of equal participating shares/units, which represent the shareholders'/unit-holders’ share of the assets and entitlement to profits or losses. Because funds are a form of collective investment that continues throughout their terms, the rights and duties of participants are defined and restricted by the common interest since they relate to third parties' rights. As such, in cases where the fund is managed on the basis of agency, the shareholders/unit-holders will waive their right to management, redemption or liquidation, except in accordance with the limitations and conditions set out in the statutes and by-laws” (AAOIFI, FAS No. 14)*.

There are different types of *Shariah*-compliant funds. The aggregate value of all outstanding Islamic funds was $108 billion which was 4% of the total asset values of the Islamic finance industry at the end of 2018. The total number of Islamic funds outstanding was 1,701 of which 176 funds were launched in 2018 (Refinitiv, 2019). The equity-based funds dominate the Global Islamic funds management. At the end of 2017, the share of these funds was 42% (592 funds outstanding) of the 1,410 funds outstanding. The second-largest asset class of Islamic funds was the mixed-asset funds with 234 funds outstanding which were equivalent to 16.6% of the total number of funds outstanding at the end of 2017. Bonds and Money market funds take the third and fourth position while the real estate funds rank last with only 23 real estate funds at the end of 2017. Out of the total values, the top three countries such as Iran, Malaysia, and Saudi Arabia shared 87% of the market value of the sector (Thomson Reuters, 2018).

### 2.3 Empirical studies on the performance of Islamic funds

Due to their unique characteristics including negative screening (exclusion), the Islamic investment funds universe developed unique risk-return characteristics that limited their exposure to the effects of the global equity markets. Studies on the relationship and comparison between the risk and return performance of Islamic funds and their conventional counterparts as along with market benchmarks have shown mixed results. However, majority of the empirical studies have not established a statistically significant difference between the economic returns reported by Islamic fund managers and their peers in the conventional market.

Table 1 presents summary of the findings of major past studies that evaluate the absolute and risk-adjusted-performance as well as riskiness of Islamic funds, SRIs, conventional funds, and market benchmarks in different geographical areas and time periods.

**Table 1:** Performance of Islamic investment funds compared with conventional funds, SRI, and the market.

Title of the Study	Author (Year)	Country	Sampling	Model Used	Major Findings
“The Islamic mutual fund performance: New evidence on market timing and stock selectivity”	Mansor and Bhatti (2011)	Malaysia	1990–2009 128 Islamic funds, 350 Conventional funds, & Kuala Lumpur Composite Index (KLCI)	Jensen Alpha version of CAPM, Treynor ratio, Sharpe ratio	- Islamic funds outperformed against conventional funds and market benchmark - Islamic fund managers had better stock selectivity performance but inferior market timing ability than conventional fund managers
“Investment in Unit Trusts: Performance of Active and Passive Funds”	Shamsher et al. (2000)	Malaysia	1995–1999 41 mutual funds	Jensen's alpha (CAPM), Treynor Ratios, Sharpe Ratio	- No statistically significant difference between the performance “actively managed” and “passively managed” funds - Both funds underperformed the market
“Investigating the Performance of Islamic Mutual Funds: Evidence from an Emerging Economy”	Abdul Rafay and Muhammad (2017)	*Pakistan*	2008–2013 KSE-30 & KMI-30 Indexes	ARCH/GARCH Models	Islamic mutual funds have consistent financial performance and risk (volatility) rate with their conventional counterparts
“Islamic mutual funds financial performance and international investment style: evidence from 20 countries”	Hoepner et al. (2011)	20 countries (from 5 regions)	1990–2009 265 Islamic & conventional funds	- One factor model, Fama and French (1993)'s 1-factor model, 3-factor model, Carhart (1997) model, 3 level Carhart model, and conditional 3 level Carhart model	- National characteristics to explain the differences in the performance of Islamic funds - In the GCC & Malaysia, Islamic Funds had competitive performance and sometimes outperformed against market benchmarks - Islamic funds significantly underperformed against their benchmarks in countries do not have well developed Islamic financial institutions or services - Especially Islamic funds from predominantly Christian economies trail their benchmarks.
“Mutual Funds Performance: Conventional and *Shariah* Product”	Agussalim et al. (2017)	Indonesia	2007–2014 IHSG), JII, SBI, and SBIS	Jensen's Alpha, Treynor index, and Sharpe index,	- There is no difference between absolute performance and volatility of Islamic and Conventional funds - Conventional funds had better risk-adjusted performance than Islamic funds - The Treynor index & Jensen's Alpha results indicated that Islamic funds had a high level of risk that their conventional peers
“Islamic Versus Conventional Mutual Funds Performance in Saudi Arabia: A Case Study”	Merdad et al. (2010)	Saudi Arabia	2003–2010 12 Islamic and 16 non-Islamic funds	Jensen Alpha (CAPM), Treynor, & Sharpe ratio	- Islamic funds underperformed against Conventional funds during the full- period & bullish period, - In Bearish & Crisis periods, Islamic funds outperformed against conventional funds
“Risk and return characteristics of Islamic equity funds”	Hayat and Kraeussl (2011)	Malaysia	2000–2009 145 Islamic Equity Funds, KLCI, & KLSI	CAPM performance Analysis & Multivariate regression model	- Islamic funds underperformed against both conventional and Islamic benchmarks - Islamic fund managers are bad market timers.
“The Performance of Islamic Equity Funds: A Comparison to Conventional, Islamic and Ethical Benchmarks”	Farid (2008)	Four Regions and Malaysia	1997–2002 46 Islamic Equity funds (IEFs) and 3 different benchmarks:	Sharpe measure, one-factor model, Fama and French (1993) 3-factor model.	- Islamic funds underperformed against their benchmarks - No statistically significant differences between ethical & Islamic fund performances
“Investigation of the performance of Malaysian Islamic unit trust funds Comparison with conventional unit trust funds”	Abdullah et al. (2007)	Malaysia	1992–2001 65 Funds (14 Islamic and 51 conventional funds)	- Jensen Alpha, Adjusted Sharpe Index,	- In bearish periods: Islamic funds outperformed against conventional funds - In the bullish period: Conventional funds outperformed
“Islamic Mutual Funds”	Elfakhani et al. (2007)	Global: Divided into regions	1997–2002 46 Islamic mutual funds, Islamic Index & Conventional Index	- Jensen's alpha, Treynor Ratios, Sharpe index, and ANOVA	- Around 29 funds outperformed against the indexes, but the result was not statistically significant
“The Investment Performance of U.S. Islamic Mutual Funds”	Climent et al. (2020)	US	1987–2018 5 Islamic funds, 20 conventional Funds, and 10 SRIs	- Jensen's alpha based on one factor CAPM and Four-factor Model	- During the period (1987–2018), Islamic funds outperformed conventional funds with comparable characteristics - However, during (2000–2018), there were no significant differences in performance - Islamic funds achieved levels of adjusted performance that did not significantly differ from those of SRI funds.
“Are Islamic mutual funds exposed to lower risk compared to their conventional counterparts? Empirical evidence from Pakistan”	Maroof (2020)	Pakistan	2009–2016 206 funds (90 Islamic & 116 conventional funds)	- single factor CAPM and multifactor models such as Fama French three factors model and Carhart four factors model are used	- Islamic funds have lower risk exposure (including total, systematic, idiosyncratic and downside risk) compared with their conventional counterparts

Generally, the results of the studies summarized in Table 1 can be categorized into three. The first group of the studies established that Islamic funds outperform non-Islamic funds and market benchmarks. Among others, Climent, et al. (2020), Mansor and Bhatti (2011), Abdullah, et al. (2007), Merdad et al. (2010), and Elfakhani et al. (2007) argued that Islamic funds showed better performance compared to their conventional counterparts and market benchmarks. Mansor and Bhatti (2011) indicated that Islamic funds perform better than conventional funds and market benchmark in Malaysia. Although Islamic fund managers have inferior market timing ability than conventional fund managers, they have better stock selectivity performance. Abdullah et al. (2007) have established that Islamic funds outperform against their conventional counterparts during bearish periods; whereas Elfakhani et al. (2007) reported that Islamic funds showed equivalent performance with market indexes.

The second group of the studies argued that there is no performance difference between Islamic and non-Islamic funds and/or market benchmarks. It means that the financial performance of Islamic funds is equivalent to SRIs and conventional funds. Among other who support this argument, Agussalim et al. (2017), Farid (2008), and Abdul Rafay and Muhammad (2017) argue that either there is no difference between the funds in terms of financial performance or the differences, if exist, are not statistically significant to justify that one fund outperformed the other and vice versa.

In the third category, there are studies that reported negative performances results against Islamic funds that Islamic funds underperform against their conventional peers. Some of these empirical studies are Hayat and Kraeussl (2011), Merdad et al. (2010), Mansor and Bhatti (2011) and Shamsher et al. (2000). In Malaysia, Islamic funds underperform against both conventional funds and Islamic benchmarks. In this market, the market timing ability of Islamic fund managers is bad (Hayat and Kraeussl, 2011). Merdad et al. (2010) have also reported that Islamic funds underperformed against Conventional funds during the bullish period. Islamic funds have also underperformed against their Islamic benchmarks, the Islamic market index (Shamsher et al., 2000).

Accordingly, we formulate the following hypotheses:

Hypothesis 1:

*H1a. Islamic funds on average underperform against SRIs and provide less risk-adjusted returns to their investors*
*H1b. Islamic funds on average underperform against conventional funds and provide less risk-adjusted returns to their investors*

Hypothesis 2:

*H2. Islamic funds on average underperform against the regional Islamic market benchmarks and provide less risk-adjusted returns to investors*

Islamic investments' compliance with *Shariah* principles takes into consideration the broader ‘ethical investment criterion’. ‘Ethical’ from an Islamic perspective implies that Islamic investments should be more concerned about other objectives that benefit society through impact investing rather than only focusing on profit-maximizing goals. The Islamic investment horizon is also limited compared to the conventional investment alternatives as *riba* (interest) related activities, investments in highly levered businesses, and prohibited goods and services are excluded from investment alternatives. The Islamic funds included in this study are only those funds that integrated SGDs in their investment policies or invested in the investment alternatives selected for this study. Accordingly, the investment scope of our sample Islamic funds is limited compared to other Islamic finance investment products. Therefore, our first and second hypotheses, H_1_ & H_2_, are based on this assumption that apart from the limited investment alternatives available for Islamic funds, they incorporate at least one of the SDGs in their investment objectives; hence, their expected returns should be lower than the SRIs and conventional funds.

In this paper, better financial performance means that the Islamic fund portfolios or their counterparts would provide a comparatively better risk-adjusted financial returns to investors. Accordingly, there will be differences in terms of the level of risks between these portfolios as risk and return have a direct relationship in theory. Based on these assumptions and the first two hypotheses, we formulated the third hypothesis, H_3,_ that the level of risks in Islamic funds is expected to be lower due to the low-risk-low-return principle and vice versa.

Hypothesis 3:

*H3a. Islamic funds have lower risk exposure compared to SRIs*
*H3b. Islamic funds, have lower risk exposure compared to conventional funds*
*H3c. Islamic funds have lower risk exposure compared to Islamic market benchmarks*

## 3 Data and methods

### 3.1 Data and sampling design

Our sampling covers four geographical areas namely North America, Asia Pacific, Europe, and the GCC regions. Based on this, the data in this study consists of a set of variables such as fund returns, Islamic market indices, conventional market indices, and sustainability indices, both at Global and regional levels were included. Table 2 presents the descriptions of the variables used in the study. Together with the Islamic funds, the time series data for the monthly average returns of SRIs (represented by Dow Jones regional sustainability index) conventional funds (represented by DJ regional index), and the monthly average of Dow Jones Islamic Market regional index were used.

**Table 2:** Description and Operation of the variables.

	Variables	Description	Market Proxy Used as a Benchmark	Data Sources
Panel A: Islamic Funds	IFA	Islamic Investment Fund Portfolio for North America. The portfolio formed using Islamic funds from USA and Canada	DJIM U.S. Titans 50 Index	Bloomberg, Eikon, Funds' webpage
	IFAP	Islamic Fund Portfolio for the Asia Pacific. The portfolio constructed using 18 Islamic funds collected from Indonesia, Malaysia, and Pakistan	DJIM Asia/Pacific Index	Bloomberg; Eikon; Funds' webpage
	IFE	Islamic Fund Portfolio for Europe formed using seven Islamic funds from Ireland, Luxembourg, and the UK.	DJIM Europe Index	Bloomberg; Eikon; Funds' webpage
	IFGCC	The GCC Islamic Funds Portfolio. It was constructed using 14 Islamic Investment funds collected from Kuwait, Qatar, and Saudi Arabia	DJIM GCC Index	Bloomberg; Eikon; Funds' webpage
	USD LIBOR	USD LIBOR Rate∗	1 Month USD LIBOR	IBORATE and FedPrimeRate.com^SM^

Panel B: SRI Funds	SRIA	Socially Responsible Investment Portfolio in the Americas. The Dow Jones regional sustainability index was used as a benchmark to measure the monthly average returns of SRI Portfolio	Dow Jones Sustainability North America Composite Index (.A1SGI)	Thomson Reuters (Eikon)
	SRIAP	Socially Responsible Investment Portfolio for the Asia Pacific region. DJ regional sustainability index is used as a benchmark to measure the performance of SRI portfolio in the Asian Pacific region	Dow Jones Sustainability Index Asian Pacific (P1SGI Index)	Thomson Reuters (Eikon)
	SRIE	Used to represent the SRI portfolio in Europe. The DJ regional sustainability index was used as a benchmark return for Socially Responsible investment funds in Europe	Dow Jones Sustainability Europe Index (.DJSEUR)	Eikon, Bloomberg
	SRIGCC	The variable represents SRI in the GCC region. Since there is no regional sustainability index for the GCC, we used the Global sustainability index as a benchmark return for SRI in the GCC region.	DJ Sustainability World Index (.W1SGI)∗∗	Thomson Reuters (Eikon)
Panel C: Conventional Funds	CIFA	Conventional Investment Funds portfolio for the Americas. The study used the Dow Jones Regional Market index for the Americas as a benchmark for Conventional funds in the region.	DJ Americas Index (.A1DOW)	Thomson Reuters (Eikon)
	CIFAP	Represents the conventional investment fund portfolio in the Asia/Pacific region. The DJ Regional Market index was used as a proxy to measure benchmark returns of conventional funds in the region	DJ Asia/Pacific Index (ˆP1DOW)	Thomson Reuters (Eikon)
	CIFE	Conventional Investment Fund Portfolio for Europe. The DJ Europe Index used as a proxy for the Regional conventional Market. The index used as a benchmark to measure average returns for conventional funds in Europe	DJ Europe Index (ˆE1DOW)	Thomson Reuters (Eikon)
	CIFGCC	Conventional Investment Fund portfolio for the GCC region. Our study used the DJ GCC market index as a benchmark to estimate the returns of conventional fund portfolio in the region	Dow Jones GCC Index (ˆDJGCC).	Thomson Reuters (Eikon)
Panel D: Islamic Markets Benchmark	IMIA	Represents the Islamic market index for the Americas. The study used the DJ regional Islamic index (.DJUS50) as a benchmark for Islamic Markets in North America region	Dow Jones Islamic Market U.S. Titans 50 Index (.DJUS50).	Thomson Reuters (Eikon)
	IMIAP	Islamic Market Index Asia Pacific. The Dow Jones Islamic Market Asia/Pacific Index was used as a benchmark for Islamic Market in the Asian Pacific region, ex-Japan	Dow Jones Islamic Market Asia/Pacific Index (.DJIAP).	Thomson Reuters (Eikon)
	IMIE	Islamic Market Index for Europe. The index was represented by the DJ regional Islamic Market Europe as a benchmark	Dow Jones Islamic Market Europe Index (.DJIEU Index).	Thomson Reuters Eikon
	IMIGCC	Islamic Market Index for the GCC. The regional DJ index (.DJIGCC Index) as a benchmark for the GCC Region Islamic Market	Dow Jones Islamic Market GCC Index (.DJIGCC Index).	Thomson Reuters Eikon

The DJ Islamic Market Indexes are used as yardsticks in evaluating the performance of Islamic funds of the appropriate category (Ghoul and Karam, 2007). Therefore, we used the Dow Jones Islamic Market regional index as a benchmark for the Islamic markets in each region. The study also used Dow Jones Global and regional market indices (as applied in Hayat and Kraeussl (2011) as market benchmarks for the selected funds, i.e. Dow Jones regional index as a proxy for conventional funds and the Dow Jones Regional Sustainability index as a proxy for SRI funds. We also used the dollar-denominated monthly average LIBOR Rate (i.e. USD LIBOR) as a proxy for the risk-free interest rate. It is common in Islamic finance research to use conventional interest rates as a benchmark in performance evaluation. Among other Reddy et al. (2017) used the daily LIBOR; and Boo et al. (2016) as well as Taib and Isa (2007) used the monthly Kuala Lumpur Interbank Offered Rate (KLIBOR) as a proxy for risk-free interest rates.

The selection of the study period and sample size were justified by the availability of data on the selected funds and market benchmarks. Due to this, the study period and sample size vary from one region to another as shown in Table 3, below. The data for Islamic funds were collected from 11 countries which were divided into four different geographical regions: the Americas, Asia Pacific, Europe, and GCC. The basis for classifying the data into these four groups was consistent with the Dow Jones regional indices for Islamic and conventional funds.

**Table 3:** Data collection period and sample size.

Geographic Area	Data Collection Period		No of Observations per Variable
	Start Period	End Period	
Americas	January - 2010	28-Feb-2019	110
Asia Pacific	April - 2010	28-Feb-2019	107
Europe	November - 2012	28-Feb-2019	76
GCC	April - 2012	28-Feb-2019	81

Our sampling process was based on the SDGs embeddedness. In the selection of Islamic funds, we take into account the funds' investment policies, asset allocation, and fund objectives. Our sample included only Islamic funds that have policies that promote the SDGs or invest in activities that directly support the achievement of the SDGs. For this purpose, we identified and used five core investment areas namely agriculture, education, health, affordable and clean energy, and clean water and sanitation as criteria to select Islamic funds. These criteria were derived from five core Sustainable Development Goals (SDGs): Goal 2 (Zero Hunger), Goal 3 (Good Health & Well-Being), Goal 4 (Quality Education), Goal 6 (Clean Water and Sanitation), and Goal 7 (Affordable and Clean Energy). The choice of these five SDGs was justified by their importance for the majority of OIC member countries and the status of their achievements under the Millennium Development Goals (MDGs)^6^ agenda.

Therefore, for an Islamic fund to be included in our sample Islamic fund portfolio, it should integrate at least one of the identified SDGs in their investment policies and fund objectives. However, due to the very limited availability of information about the investment policies of the majority of the Islamic funds, we assessed the funds' areas of investment and sector focus. Accordingly, we limited our sample only on those funds that either take into consideration the above mentioned five SGDs in their investment decisions or invest their money in the above-mentioned investment areas (sectors). It is also easier to achieve consistency when calculating returns if only open-end funds are considered (Climent et al., 2020). Thus, we could identify only 41 open-ended funds and categorized them into four groups according to their geographical location. We formed a portfolio representing each region.

The funds recorded in the Thomson Reuters Eikon and Bloomberg database were screened using the funds' country of domicile. The sample size was designed based on monthly average data on each variable from January 2010 to February 2019. The reason for selecting this range of time was that it allows us to investigate the post-crisis performance of the funds, i.e. after the 2008/09 financial crisis. Due to the unavailability of an equal amount of data for all regions, the time series of the data could not start from the same period. Hence, for the Americas, the study period started from 2010 and the final sample size includes 110 observations per variable, i.e. a total of 440 observations. In the other regions, the total number of observations is 428 (Asia Pacific), 304 (Europe), and 324 (GCC). Based on this, the aggregate number of observations, excluding interest-free rates and independent variables, is 1,496.

### 3.2 Econometric models

#### 3.2.1 Absolute return measurements

The absolute return (return relative to the investment itself) measurement is based on the following model (Eq. (1)):

(1) $$R_{p}=\frac{1}{n}\sum_{i=1}^{n}R_{pt}$$

Where:

$R_{p}$*is the absolute rate of return on a portfolio p;* $R_{pt}$ *is Return on portfolio p in time t; n is the number of observations.*

The monthly average returns of the funds were calculated using the Net Asset Value (NAV) of the funds at the beginning and end of the month. Since dividend payment decreases the NAV of the fund, the new value at the end of the period may not indicate the actual returns (performance) of the fund. Therefore, to overcome this limitation, we added back dividends paid between the beginning and end of the period. Hence, the periodical (monthly) total absolute returns of the portfolios (funds) were calculated by using the following formula:

(2) $$R_{p,\;t}=\frac{D_{t}+\left(NAV_{t}-NAV_{t-1}\right)}{NAV_{t-1}}$$

*Where*: $R_{p}$ *Return on a portfolio; D*_t_ *is Dividend paid in time t, and NAV is Net Asset Value*.

#### 3.2.2 Risk-adjusted performance measurements

The risk-adjusted performance is based on the absolute risk-adjusted performance measure (Sharpe Ratio) and the relative risk-adjusted performance measures (Treynor Ratio and Jensen's alpha using CAPM). The main justification for choosing these models is that their underlying theories have been heavily examined and remain well-established following various empirical tests that have been undertaken in the past to validate the effectiveness of these models. Due to their simplicity, these models are more popular among both practitioners and academics. These traditional portfolio-performance valuations are the most widely applied models in various empirical studies including in Abdullah et al. (2007), Schröder (2007), Statman (2006), Chong et al. (2006), Bello (2005), Hussein and Omran (2005), Kreander et al. (2005), Shah Zaidi et al. (2004), Yaacob and Yakob (2002), Sauer (1997), and Mallin et al. (1995).

1. Sharpe Ratio

The Sharpe ratio was first introduced by Sharpe (1966) to evaluate the performance of mutual funds. It is now widely accepted and enjoys almost ubiquitous implementation in the finance world (McLeod and Vuuren, 2004). It has applied in many studies to evaluate how well portfolios perform compared to a risk-free investment (Frank and Brown, 2012, p.65). We followed the basic rule that if the value of the index is unbiased, the higher the Sharpe ratio, the better the performance of a portfolio and vice versa.

(3) $$S_{p}=\frac{r_{p}-r_{f}}{\sigma_{p}}$$

Where: $S_{p}$ is Sharp Ratio of portfolio p; $r_{p}$ is the portfolio's return; $r_{f}$ is the risk-free rate of return proxied by the one month USD LIBOR; and $\sigma_{p}$ is the standard deviation of portfolio p.

2. Treynor Ratio (Index)

Unlike the Shape ratio, the Treynor ratio (Treynor, 1965) evaluates the portfolio's performance against a different benchmark and it indicates how well a portfolio outperforms the whole market rather than the risk-free rate of return (Frank and Brown, 2012, p.63). The role of the ratio in this paper was to

(4) $$T_{p}=\frac{r_{p}-r_{f}}{\beta_{p}}$$

*Where:* $T_{p}$ *is Treynor ratio of a portfolio; and* $\beta_{\rho }$ *is Portfolio's Beta*.

3. Jensen's Alpha using CAPM

The Jensen's Alpha (Jensen, 1968) represents the average rate of return on a portfolio above or below the return predicted by the Capital Asset Pricing Model (CAPM) for a given portfolio's beta and the average market return. It is the intercept of the regression equation in the CAPM and is in effect the excess return adjusted for systematic risk (Bacon, 2008, p.72). It is determined using the following model, CAPM (Eq. 5):

(5) $$Alpha\;\left(\alpha \right)=r_{p}-\left[r_{f}+\beta_{p}\left(r_{m}-r_{f}\right)\right]$$

*Where:* $r_{p}$ *is Expected Portfolio Return;* $r_{f}$ *the mean return on a risk-free proxy (in this case monthly average USD LIBOR rate);* $\beta_{p}$ *is Beta of the Portfolio; and* $r_{m}$ *is Expected Market Return*.

The sign of the Alpha indicates the performance of the investment relative to the market. A positive Alpha interpreted as an indication of a good performance. This means a portfolio manager has "beaten the market" with his fund selection and portfolio formation skills (Brooks, 2008, p.113). Therefore, using the above Jensen's measure, we can determine whether the portfolios are earning the proper return for their level of risk. The parameter indicates whether our funds underperform or outperform their benchmarks (market index).

## 4 Results and discussions

### 4.1 Descriptive statistics

**Figure 1:**
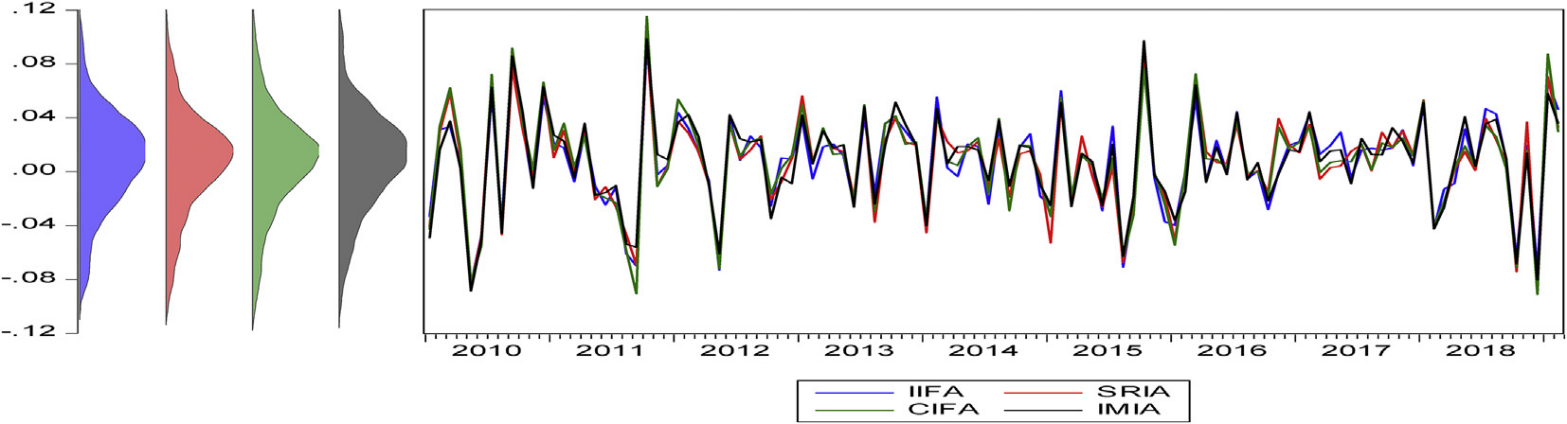
Monthly returns of the funds' portfolios and the Islamic market index in the Americas.

**Figure 2:**
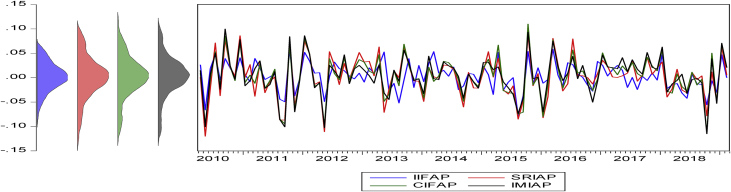
Monthly return of fund portfolios and Islamic market in the Asia Pacific.

**Figure 3:**
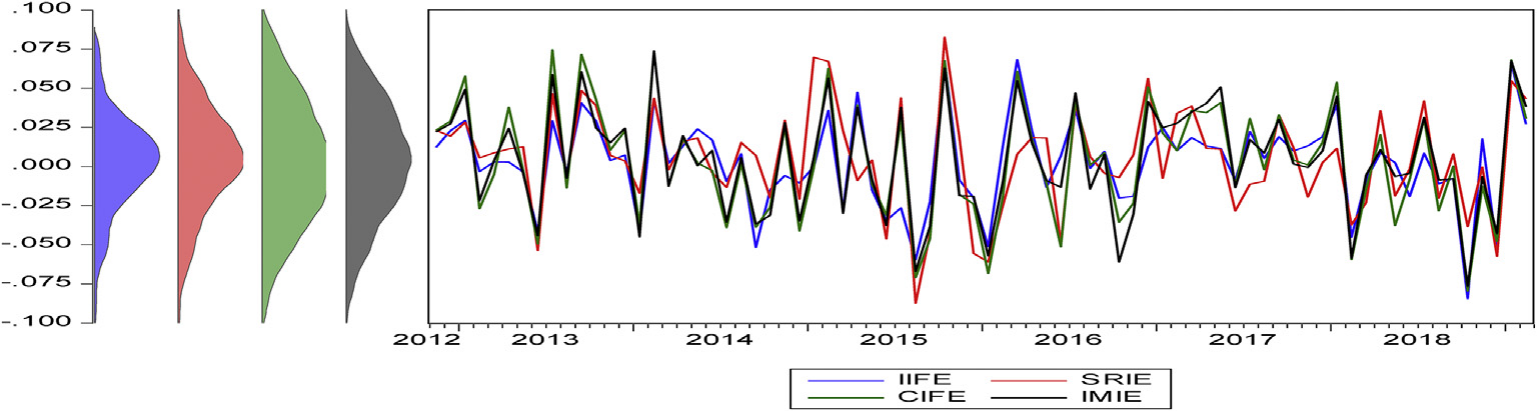
Monthly return of fund portfolios and Islamic market in Europe.

**Figure 4:**
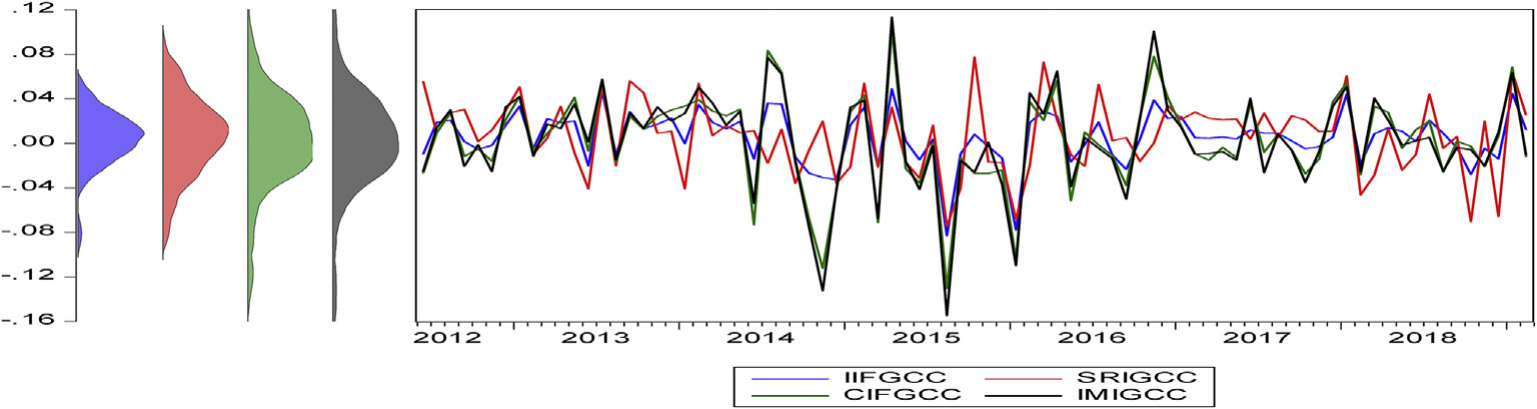
Monthly return of fund portfolios and Islamic market in the GCC region.

The statistical proprieties of the data are presented in Table 4. The numerical values show the historical behavior of the time series data used in the analysis. The table reports the descriptive statistics of the variables in five panels. For example, Panel (A) reports the basic statistical descriptions for the monthly returns of the four variables in the Americas regions (see Figure 1). Figure 1, Figure 2, Figure 3, Figure 4 summarize the monthly weighted average returns of the fund portfolios and the Islamic markets in the four regions.

**Table 4:** Statistical description of variables.

Region	Variables	N	Mean (μ)	Std. D. (σ)	Max.	Min.
Panel A: Americas Region	IFA	110	0.007706	0.034997	0.0955	-0.0837
	SRIA	110	0.007312	0.035945	0.0993	-0.0892
	CIFA	110	0.008069	0.037469	0.1154	-0.0911
	IMIA	110	0.008372	0.035317	0.0988	-0.0886
Panel B: Asia Pacific Region	IFAP	107	0.00303	0.028329	0.0584	-0.0789
	SRIAP	107	0.001874	0.042221	0.0886	-0.1199
	CIFAP	107	0.003507	0.041579	0.11	-0.1137
	IMIAP	107	0.004153	0.040977	0.0997	-0.115
Panel C: Europe	IFE	76	0.002736	0.028917	0.0684	-0.0843
	SRIE	76	0.005134	0.032956	0.0826	-0.0873
	CIFE	76	0.00352	0.037588	0.0747	-0.0795
	IMIE	76	0.004464	0.034873	0.0739	-0.0764
Panel D: The GCC Region	IFGCC	81	0.005373	0.023374	0.0488	-0.0831
	SRIGCC	81	0.006694	0.033439	0.0778	-0.0743
	CIFGCC	81	0.002978	0.041355	0.1029	-0.1303
	IMIGCC	81	0.002502	0.044113	0.1133	-0.1549
Risk-Free Rate of Returns	USD LIBOR∗	110	0.00582	0.006575	0.0251	0.0015
	USD LIBOR∗∗	107	0.005918	0.006641	0.0251	0.0015
	USD LIBOR∗∗∗	76	0.007308	0.007451	0.0251	0.0015
	USD LIBOR∗∗∗∗	81	0.007	0.007315	0.0251	0.0015

Note: The USD LIBOR rate is based on 110 (∗), 107(∗∗), 76(∗∗∗), and 81(∗∗∗∗) monthly observations respectively.

### 4.2 Correlation analysis

Correlations between different asset classes need important consideration in asset allocation and portfolio formation. Table 5 shows the pairwise correlation between the variables within each region. The correlation results, as shown in Table 5, reveal strong and positive correlations between the variables.

**Table 5:** PAIRWISE correlation matrix analysis.

AMERICAS					ASIA PACIFIC				
	IFA	SRIA	CIFA	IMIA		IFAP	SRIAP	CIFAP	IMIAP
IFA	1.0000	0.9424	0.9596	0.9694	IFAP	1.0000	0.6863	0.7337	0.7402
SRIA		1.0000	0.9823	0.9511	SRIAP		1.0000	0.9534	0.9091
CIFA			1.0000	0.9546	CIFAP			1.0000	0.9633
IMIA				1.0000	IMIAP				1.0000
EUROPE					GCC REGION				
	IFE	SRIE	CIFE	IMIE		IFGCC	SRIGCC	CIFGCC	IMIGCC
IFE	1.0000	0.6472	0.8550	0.8711	IFGCC	1.0000	0.6626	0.8826	0.8804
SRIE		1.0000	0.7925	0.7635	SRIGCC		1.0000	0.3739	0.3556
CIFE			1.0000	0.9665	CIFGCC			1.0000	0.9817
IMIE				1.0000	IMIGCC				1.0000

A visual inspection of Table 6 affirms that correlations among the funds in the Asia Pacific, Europe, and the GCC regions were moderate during the period except the high correlation between some of the variables. In general, the degrees of the correlation between the variables ranged from a strongly positive (0.9665 in Europe between conventional funds and Islamic markets) to a moderately positive relationship (0.3556 between SRIs and Islamic markets in the GCC). As correlations are used as a measure of the diversification of a portfolio, the overall results obtained from the descriptive and correlation analysis are important to conduct the performance analysis.

**Table 6:** Absolute returns mean difference test results in Americas and Asia pacific geographic areas.

	North America			Asia-Pacific		
	Mean	Mean Difference	Correlation	Mean	Mean Difference	Correlation
Islamic	0.007706	0.0003945 (0.343) [0.733]	0.942 (0.000)	0.003030	0.0011561 (0.389) [0.3222]	0.686 (0.000)
SRI	0.007312			0.001874		
Islamic	0.007706	-0.0003627 (-0.359) [0.720]	0.960 (0.000)	0.003030	-0.0004766 (-0.174) [0.862]	0.734 (0.000)
Conventional	0.008069			0.003507		
Islamic	0.007706	-0.0006655 (-0.803) [0.424]	0.969 (0.000)	0.003030	-0.0011234 (-0; 421) [0.675]	0.740 (0.000)
Islamic Market Bench.	0.008372			0.004153		
SRI	0.007312	-0.0007573 (-1.122) [0.264]	0.982 (0.000)	0.001874	-0.0016327 (-1.319) [0.190]	0.953 (0.000)
Conventional	0.008069			0.003507		

N.B.: ( ) denotes t-statistics and [ ] denotes p-values.

### 4.3 Results of performance analysis

To determine the financial performance divergences between the Islamic funds and their counterparts, we used both the absolute return and the risk-adjusted return analysis.

#### 4.3.1 Absolute return analysis

The absolute return analysis was carried out in two stages: within the geographical area and between the different geographical regions. In the first stage, the absolute return characteristics of all fund portfolios were compared with each other and with the Islamic market benchmarks within the funds' geographical areas. In the second stage, the absolute financial performance (returns) of Islamic fund portfolios compare each other across the regions. For both cases, we applied the paired-test method to compare the absolute financial performance of the portfolios based on the monthly mean return values to test the first two hypotheses; i.e. *H*_*1*_ *and H*_*2.*_

Table 6 presents the mean differences of the monthly average absolute returns of the funds' portfolios in the Americas and Asia Pacific geographic categories. The results indicate that there is no statistically significant difference between absolute mean returns of the funds in both categories at 5% significant level. Islamic funds on average showed better performance compared to SRI in both regions. However, conventional funds performing well compared to both Islamic funds and SRIs in both regions. None of the results are statistically significant. Therefore, based on the results obtained from Table 6, the hypotheses (H_1_ and H_2_) that Islamic funds underperform against other funds and the Islamic market benchmark have rejected in both regions. There is no statistically significant evidence to accept the null hypothesis and establish that Islamic funds have less absolute performance compared to their conventional counterparts as well as the market benchmarks.

Similarly, Table 7 presents the absolute performance test results for Europe and the GCC regions. The test results presented in Table 8 also indicated that there is no statistically significant difference between the mean return values of the three funds in both regions. In Europe, the absolute performance of Islamic funds was less well during the period compared to the other funds and the Islamic market benchmark. Nevertheless, in the GCC region, the performance of Islamic funds was relatively better than conventional funds and the regional Islamic market index. However, none of the results are statistically significant. Therefore, based on the results presented in Table 8, the two null hypotheses, i.e. H_1_ and H_2_, are not true and have rejected.

**Table 7:** Non-risk-adjusted absolute returns mean difference test results in Europe and the GCC geographic areas.

	EUROPE		GCC REGION			
	Mean	Mean Difference	Correlation	Mean	Mean Difference	Correlation
Islamic	0.0027355	-0.00239868 (-0.797) [0.428]	0.647 (0.000)	0.005373	-0.001324 (-0.471) [0.639]	0.663 (0.000)
SRI	0.0051342			0.006694		
Islamic	0.0027355	-0.00078421 (-0.346) [0.730]	0.855 (0.000)	0.005378	0.0023995 (0.971) [0.360]	0.883 (0.000)
Conventional	0.0035197			0.002979		
Islamic	0.0027355	-0.00172895 (-0.877) [0.383]	0.871 (0.000)	0.005378	0.002876 (0.994) (0323)	0.880 (0.000)
Islamic Market Bench	0.0044645			0.002502		
SRI	0.0051342	0.00161447 (0.608) [0.545]	0.792 (0.000)	0.006689	0.0037106 (0.788) [0.433]	0.374 (0.000)
Conventional	0.0035197			0.002979		

N.B.: ( ) denotes t-statistics and [ ] denotes p-values.

**Table 8:** Absolute (Non-Risk-Adjusted) Performance Test Results of Islamic Funds' Portfolios in the four Geographic Areas.

	Mean	Mean Difference	Correlation
IFA	0.009108	0.0079526∗∗ (2.456) [0.016]	0.533 [0.000]
IFAP	0.001155		
IFA	0.009108	0.0063724∗∗∗ (3.028) [0.003]	0.814 [0.000]
IFE	0.002736		
IFA	0.009108	0.0037276 (1.282) [0.204]	0.598 [0.000]
IFGCC	0.005380		
IFAP	0.001155	-0.0015803 (-0.604) [0.548]	0.671 [0.000]
IFE	0.002736		
IFAP	0.001155	-0.0042250 (-1.449) [0.152]	0.511 [0.000]
IFGCC	0.005380		
IFE	0.002736	-0.0026447 (-1.065) [0.290]	0.659 [0.000]
IFGCC	0.005380		

N.B.: ( ) denotes t-statistics and [ ] denotes p-values; ∗∗ denotes H_0_ rejected at 5 percent level of confidence; ∗P < 0.01; ∗∗P < 0.05; ∗∗∗P < 0.001.

In the second stage of performance evaluation, we conducted a test to check the existence of any absolute performance differentiation among the Islamic fund portfolios across the regions. The absolute return pair-tests results, as reported in Table 8, indicate that Islamic funds in the Americas (IFA) significantly outperformed against Islamic funds in Europe (IFE) and the Asia Pacific regions (IFAP). Although IFA also showed better performance compared to the IFGCC during the same period, the results were statistically insignificant. Islamic funds in the Asia Pacific region were the least performing assets compared to the Islamic funds in the other regions.

#### 4.3.2 Risk-adjusted performance test analysis

Many researchers argue that apart from absolute mean-return differences in fund performance, factors such as exposure to risk may impact fund returns. It is important to measure the amount of risks involved in making the absolute return. Thus, once absolute return analysis is carried out, it is then a logical evolution to undertake a comparative analysis after controlling for differences in their risk profile (Reddy et al., 2017). We conducted the risk-adjusted performance comparison using the Sharpe ratio and the Traynor ratios.

Table 9 presents the results of the risk-adjusted performance test results. From the results demonstrated in the table, it is clear that Islamic funds have a relatively higher Sharpe ratio only compared to the SRIs in the Americas. It indicates that on a risk-adjusted basis, the Islamic funds outperform the SRIs. However, the Islamic funds showed less performance in terms of excess return per unit of risk measurements against the conventional funds and the regional Islamic market benchmark, which have a Sharpe ratio of 5.87% and 7.09% respectively.

**Table 9:** Sharpe ration and Portfolio riskiness analysis in the Four Regions.

Region	Portfolio	Risk-Adjusted Measure		Absolute Risk Measures	
		Sharpe	Treynor	Coefficient of Variation	Standard Deviations
Americas	Islamic	0.053195	0.001958	4.543612	3.50%
	SRI	0.041147	0.001914	4.915909	3.59%
	Conventional	0.058719	0.002395	4.647339	3.75%
	Islamic Market	0.070946	0.002893	4.217356	3.53%
Asia Pacific	Islamic	-0.095734	-0.005644	9.345743	2.83%
	SRI	-0.094182	-0.004520	22.518273	4.22%
	Conventional	-0.057258	-0.002494	11.870266	4.16%
	Islamic Market	-0.042373	-0.001823	9.850446	4.10%
Europe	Islamic	-0.151919	-0.006332	10.570174	2.89%
	SRI	-0.063354	-0.002924	6.416232	3.29%
	Conventional	-0.097907	-0.003516	10.673971	3.76%
	Islamic Market	-0.079477	-0.002783	7.807681	3.49%
GCC	Islamic	-0.066678	-0.003480	4.344848	2.34%
	SRI	-0.009049	-0.000313	4.998954	3.34%
	Conventional	-0.096883	-0.008300	13.882694	4.14%
	Islamic Market	-0.101199	-0.010373	17.605381	4.41%

In the remaining three regions, the risk-adjusted test results show negative Sharpe ratios for all funds and the Islamic market benchmarks. Table 9 demonstrates mixed results. In the Asia-pacific region, Islamic funds have an equivalent performance with the SRIs; but both funds underperform compared to the conventional fund and the Islamic market benchmarks. On a risk-adjusted base, the results indicate that funds in these regions did not perform even equal to the risk-free rate of return during the analysis period. In the GCC region, Islamic funds have a relatively better performance than the other funds and the market benchmark. However, in Europe, SRI funds showed a relatively better performance than the other funds and the Islamic market index.

A similar pattern of results was obtained in the Treynor ratios tests. All the three funds and the Islamic market benchmarks have positive Treynor ratios only in the Americas region which are similar to the Sharpe ratios. In the Americas, Islamic funds generate better value compared to SRIs. However, the Treynor ratios of Islamic funds and SRI were lower compared to the conventional fund portfolio and the Islamic market regional index during the study period both in the Americas and the Asian pacific. In the Asia Pacific region, Islamic funds generate inferior value compared to the other funds and the market benchmark. In this market, conventional funds showed a relatively better performance compared to SRI funds. However, compared to all funds, the Asia Pacific regional Islamic market had superior performance. In general, the results obtained indicate that none of the funds generate positive Treynor values at a given level of market risks in all regions except the Americas.

We also conducted a test to evaluate the risk per reward characteristics of the funds using the coefficient of variation. Table 9 presents the coefficient of variation test results which measures the funds' riskiness per unit (dollar) of reward. This is an important finding in understanding the funds' volatility per unit of return earned. The result demonstrates that Islamic funds have the least risk-reward variation ratio in all regions except in Europe. The coefficient of variation of Islamic funds in the Americas (4.54), Asian Pacific (9.35), and the GCC (4.34) is the least compared to their counterparts. The stability of returns can give more confidence to risk-averse investors. Thus, from risk-averse investors' perspective, Islamic funds represent a better investment alternative compared to other funds as they provide the most optimal risk-to-reward ratio with low volatility. The result is consistent with the third hypothesis, H_3_, that Islamic funds are less risky (volatile) than their peers and market benchmarks in all regions.

#### 4.3.3 Jensen's alpha with CAPM test result

Tables 10 and 11 present the results of the Jensen's alpha with CAPM tests together with other test results such as Beta and Adjusted R-square.

**Table 10:** Relative Risk-Adjusted Performance measurement Results in Americas and Asia Pacific Regions.

Region	Portfolio	Alpha	Beta	Adjusted R Square
AMERICAS	Islamic	-0.00054745 (-0.668) [0.506]	0.95375535∗∗∗ (41.778) [0.0000]	0.941
	SRI	0.002658∗ (1.897) [.0605]	0.784708∗∗∗ (23.823) [0.0000]	0.839
	Conventional	0.0024188∗∗∗ (2.666) [0.0089]	0.9379110∗∗∗ (40.563) [0.000]	0.938
	Islamic Market	0.0019818∗ (1.857) [.0660]	0.8867971∗∗∗ (31.919) [0.0000]	0.903
ASIA PACIFIC	Islamic	-0.0019302 (-.990) [.324]	0.5427613∗∗∗ (11.533) [0.000]	0.555
	SRI	-.0026610 (-1.428) [.1561]	0.9028547∗∗∗ (20.556) [0.0000]	0.799
	Conventional	-.0019978 (-1.133) [.260]	0.9700645∗∗∗ (21.505) [0.000]	0.813
	Islamic Market	-.00222988 (-1.254) [.213]	0.96881798∗∗∗ (20.898) [0.000]	0.804

N.B.: ( ) denotes t-statistics and [ ] denotes p-values; ∗∗ denotes H_0_ rejected at 5 percent level of confidence; ∗P < 0.01; ∗∗P < 0.05; ∗∗∗P < 0.001.

**Table 11:** Relative Risk-Adjusted Performance measurement Results in Europe and the GCC Regions.

Region	Portfolio	Alpha	Beta	Adjusted R Square
EUROPE	Islamic	-0.0024717 (-1.479) [0.143]	.7387869∗∗∗ (15.758) [0.000]	0.767
	SRI	-0.0007782 (-0.313) [0.755]	.7685418∗∗∗ (10.694) [0.000]	0.602
	Conventional	-0.0026055 (-1.374) [0.173]	1.0751675∗∗∗ (18.375) [0.000]	0.818
	Islamic Market	-0.0025910 (-1.491) [0.140]	1.0204026∗∗∗ (18.555) [0.000]	0.821
GCC	Islamic	0.00056727 (0.431) [0.668]	.47813839∗∗∗ (16.076) [0.000]	0.761
	SRI	0.0000000	1.0000000	1.000
	Conventional	-0.0044539 (-1.046) [0.299]	.4927793∗∗∗ (3.679) [0.000]	0.134
	Islamic Market	-0.0052421 (-1.122) [0.265]	.4476977∗∗∗ (2.977) [0.004]	0.088

N.B.: ( ) denotes t-statistics and [ ] denotes p-values; ∗∗ denotes H_0_ rejected at 5 percent level of confidence; ∗P < 0.01; ∗∗P < 0.05; ∗∗∗P < 0.001.

The Jensen's alpha (CAPM) test results reported in Tables 10 and 11 are mixed. The results show that concerning relative performance or Jensen's alpha, all funds and the Islamic markets have negative alpha values; it implies that they do not outperform their benchmarks in Europe and the Asia Pacific. Islamic funds also underperform against their benchmark in the Americas. In the Americas, conventional funds show superior performance over their benchmarks; these indicates that investment selection skills of conventional fund managers is superior and it delivers superior risk-adjusted returns to investors. Although the figures in the table show that Islamic funds failed to beat the return given by the market in both regions, the results are not statistically significant. The SRI funds and the Islamic market performed well compared to their benchmarks in the Americas in risk-adjusted (Jensen's Alpha) at a 10% level of confidence. The results show that none of the funds outperformed their benchmarks on a risk-adjusted basis in the Asia Pacific region. Therefore, based on the results, we rejected the second hypothesis, H_2_, that Islamic funds underperform against their benchmarks in the Americas and the Asia Pacific regions. There is no statistically significant evidence to support the null hypothesis.

Although in Europe and the GCC regions, as shown in Table 11, the Jansen's Alpha figures tell that all the three funds types and the Islamic market underperform against their respective benchmarks, none of the results are statistically significant. Islamic funds in the GCC relatively outperform their benchmark but the results are not statistically significant. Hence, based on this result we rejected the H_2_ that Islamic funds have significantly underperformed compared to their benchmarks during the study period.

Regarding systematic risks, Tables 10 and 11 also present the sensitivity of the funds to their benchmarks for all regions. The figures reported in the tables show that Islamic funds in all regions except the Americas tend to be less significantly exposed to the systematic risks compared to conventional funds and the SRI. In these regions, Islamic funds are less sensitive to systematic risks or a possible change in their benchmarks. Our results support the third hypothesis, H3_a,_ H3_b_, H3_c_, that Islamic funds have less exposure to systematic risks compared to their counterparts.

The values of adjusted R-squared (R^2^) reported in Table 10 indicate that a significantly high percentage of the funds' return movements in the two regions (Americas and the Asia Pacific) are closely aligned with or explained by movements in their benchmark index. In the Americas, 94% of Islamic funds' movements explained by the movements in the regional Islamic Market index. In the Asia Pacific region, 55.5% of the Islamic funds' movements are explained by their market benchmarks, *i.e. the Dow Jones Islamic Market Asia Pacific Index.* The results of the adjusted R-squared also illustrate that changes in the Islamic fund performance in Europe and the GCC region are linked to and explained by their benchmarks. The result indicates that 76.7% of the changes made on Islamic funds in Europe and the GCC explained by the changes in the respected regional Islamic market index. In the GCC, however, conventional funds and the Islamic market have very small adjusted R-Squared values. The figure shows that only 13.4% of the changes in conventional funds were reflected by the Dow Jones GCC index which was used as a proxy for the GCC conventional markets.

#### 4.4 The synergy between Islamic Funds and SRIs to mitigate the SDGs financing gaps

Several fund managers and impact investors understand the importance and potential of SRIs to mitigate the SDGs financing gap; thus, it made the concept unavoidable (Luc et al., 2008). Islamic funds and SRIs have close similar features as both investments aim to improve the betterment and wellbeing of society. They take into account both personal values and societal concerns in investment decisions. The tenets of *Shariah*, which also used to govern Islamic economic activities, emphasize ecology, sustainability, social justice, labor rights, kindness, etc. Thus, there is a significant overlap between SRI principles and the objectives of Islamic principles or *Maqasid-al-Shariah*^7^*.*

As it is clearly understood from their definitions, there is a clear and logical fit between the focus of SRIs and the objectives of Islamic *Shariah* (*Maqasid al-Shariah*). It was also highlighted in the literature that Islamic finance is a popular form of SRI and SRI has its place in Islam (Zahari and Shanmugam, 2009). However, the objectives of Islamic finance are much bigger than the objectives of SRIs. Van and Reza (2010) stated that for SRI it is all about the rise of ‘soft’ values like human rights, environment, social rights, etc. However, for Islamic finance, it is mainly about the rise of Islamic identity. Some researchers criticize adopting SRIs principles in *Shariah*-compliant investments. It was rarely analyzed in the literature that SRIs are incompatible for widening the Islamic prospective investor base into SRIs, thus, there is no need to follow SRIs principles and guidelines in Islamic investment decisions (Stanley and Jaffery, 2009, p. 257).

Some of their main differences include the prohibition of some activities, products, and financing instruments in Islam funds but acceptable in SRIs. Unlike SRIs, it has prohibited for Islamic funds, for example, to invest in or finance certain goods and activities which considered as unlawful (*Haram*) in Islam such as producing pork meat, investing interest-based financial institutions, doing business in gambling industries, investing in the pornography industry, and business entities with high leverage (Farid, 2008). Thus, Islamic finance in general uses *Shariah* screening criteria to enable investors to ensure that the sectors and business activities they invest in are not against any *Shariah* principles (Khatkhatay and Nisar, 2006).

On the other hand, Islamic funds and SRIs have many common characteristics and some of them are presented in Table 12. The exclusion strategy is one of the similarities between *Shariah*-compliant investments and SRI's as the latter uses negative screening strategies. The ultimate goal of the two is to attain the wellbeing of society. In Islamic finance, it is believed that the wellbeing of society can be achieved only in compliance with *Shariah* principles. Advocating and financing the SDGs is one way of enhancing the society's wellbeing. Aligning legal compliance with economic objectives as well as social and environmental impacts is important to achieve the ultimate objective of Islamic law i.e. promoting well-being and preventing harm. These are also the key pillars of sustainable development. Hence, adopting various strategies and principles, including SRI principles, to achieve sustainable development in society is in line with *Shariah* objectives (*Maqasid-Al-Shariah*). Similarly, in the case of SRIs, the achievement of the betterment or wellbeing of the society requires a sustainable economic system that takes into consideration SDG objectives (Miglietta and Forte, 2011). These and other close similarities between Islamic funds and SRIs suggest the possible synergy between the two. investment universes, paving the way for the creation of a new asset class that is SRI and *Shariah*-compliant.

**Table 12:** Similarities and differences between Islamic investment funds and SRIs.

Characteristics	Islamic Investment Funds	Socially Responsible Investments
Broad Objectives (ethical, social and financial objectives)	Yes	Yes
Negative screening (filtering) criteria	Yes	Yes
Industry (Sector) exclusion	Yes (industries don't compliant with Islamic values)	Yes (Industries do not compliant with SRI criteria)
Supervision committee	Yes, “*Shariah* Supervisory Board”	Yes, “Ethical Committee”^8^
Sources of “Guidance”	*Shariah*	Universally recognized norms and standards
Binding decisions of the “Supervisory Committee”	Yes	No (their opinions have the status of advice)
Restriction on investment	Yes, (no investment in the fixed income instruments: bonds, CDs, some derivatives, preferred stocks, warranty)	No, (no restriction as far as the stocks chosen are adhere to SRI principles)
Clear definition of action limits	Yes, the guide is the Qur'an, integrated when possible with legal interpretations.	No, a universally recognized definition of social responsibility does not exist.
Income Purification process	Yes	No
Screening according to CSR, ESG, SDGs, etc.	It is not mandatory but possible to add	Yes
Best-in-Class	No, there is only distinction between permissible and prohibited (possible to add)	Yes^9^

**Figure 5:**
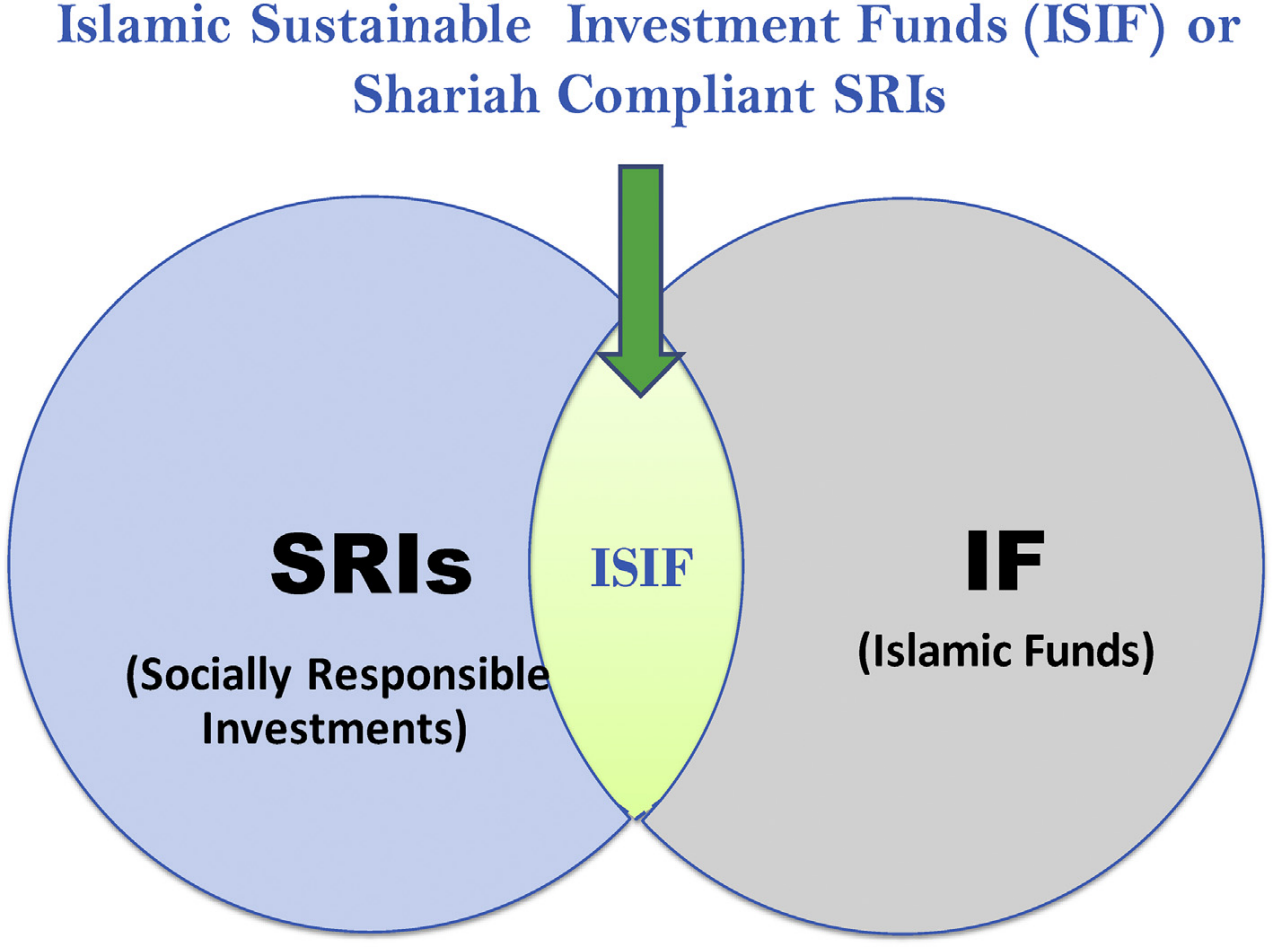
The synergy between Islamic Funds and SRIs.

Although it is a religious duty for Muslims to conduct business and investment in a more socially and environmentally responsible manner, it is not enough what is actually being done (Brugnoni, 2009). Therefore, the fusion between the two is important to optimally utilize the existing Islamic funds and other instruments to finance the SDGs, particularly in Muslim countries. The synergy widens the SRIs prospective investor base into Islamic funds. Van and Reza (2010) reported that the synergy leads to the creation of *Shariah*-compliant SRIs which ensures better financial returns to investors, makes the sector more sustainable, and positive impacts on society and the environment. The new asset class, i.e. *Shariah*-compliant SRIs, therefore, will have the characteristics of both Islamic funds and SRIs. It integrates sustainability objectives while meeting *Shariah* requirements. Thus, it can play a significant role in increasing the investor base and providing sustainable financing for the SGDs especially in Muslim countries where SRI assets are very limited (see Figure 5).

It is also equally important to work on the effectiveness of the synergy. The new asset class combining both strategies requires dedicated guiding principles and screening approaches. The screening methods, therefore, could integrate SRI principles that do not contradict *Shariah* into the existing *Shariah* screening criteria. However, the successful implementation of the new asset class requires the support of various stakeholders including policymakers, financial institutions, *Shariah* scholars, asset managers, and investors. Policymakers and regulatory bodies should promote the development of the ISIF new asset class by implementing relevant taxonomies, policies, and guidelines. These should be clearly understood and promoted by *Shariah* scholars. Further, there should be a proper framework to investigate the implementation of SRI principles. Van and Reza (2010) suggested the need for an audit process that would have to explain in detail how and why a financial product satisfies the requirements of SRI and *Shariah*. The process should be transparent and made available to all parties, especially to impact investors.

## 5 Conclusion

The achievement of the SDG agenda requires significant investments globally. According to UNCTAD, achieving the bold SDGs requires annual investments between $5 trillion to $7 trillion with an investment gap of about $2.5 trillion in developing countries. This gap cannot be filled by governments alone and requires the private sectors' active participation. Investment funds that take into account ethical objectives have the potential to mitigate the SDG financing gaps. Socially responsible investment (SRIs) funds and the morally responsible investment (MRIs) funds or Islamic funds represent two growing segments of impact investing globally. One of the two main objectives of this study was to explore synergies between the two in order to mobilize additional investments for the SDGs by targeting a wider investor base consisting of both Islamic and impact investors.

Since both Islamic funds and SRI have common objectives and investment strategies, it was interesting to observe whether they report any differences in their financial performance. Thus, the second objective of the study was to investigate the risk and return characteristics of Islamic funds mainly in comparison with SRIs in light of the SDGs. The study was based on data collected from 11 countries grouped into four geographical regions. The comparison also included conventional funds and Islamic market benchmarks. Therefore, our analysis leads to the following three conclusions: First, the absolute (none-risk adjusted) performance test results indicate that Islamic funds outperform the SRIs and underperform against conventional funds and the Islamic market benchmarks in most of the regions. But none of the results were statistically significant. Therefore, we rejected the H_1_ and H_2_ that Islamic funds underperform against their counterparts. These findings are in line with findings reported by Mansor and Bhatti (2011), Hoepner, et al. (2011), and Elfakhani et al. (2007). Second, the relative risk-adjusted performance test outcomes show mixed results. Our results established that Islamic funds have a better return per unit of risks in most of the region compared to SRIs. However, Islamic funds in Europe exhibited poor performance compared to both the SRIs, conventional funds, and the Islamic market. These results, in one way or another, are similar to the results of Hoepner et al. (2011), Agussalim, et al. (2017), Elfakhani, et al. (2007) and Farid (2008). Third, the riskiness (volatility) test results indicate that Islamic funds give the least volatile average return compared to the other funds and the market benchmark in all regions. The evidence leads us to reject H_3_ and conclude that Islamic funds are low-risk asset classes compared to the other funds and the Islamic market benchmark. A similar conclusion was reached by Agussalim et al. (2017) and Abdul Rafay and Muhammad (2017).

Although the outputs of this study demonstrate the mixed performance of Islamic funds compared to their counterparts, we believe that the inconsistency would be due to different factors such as geographical differences, the performance of fund managers, maturity of the market, etc. Hence, it is hard to conclude that Islamic funds are significantly outperforming or underperforming against other funds. The main conclusion that can be drawn is that being subject to faith-based screening (*Shariah*-based screening) and integrating SDG considerations in their investment policies; the risk-return characteristics of Islamic funds are not significantly different from their conventional counterparts and the market benchmarks. These conclusions are in one or another way consistent with the findings of a recent study by Reddy et al. (2017) and Boo et al. (2016) who have also argued that there is no significant difference between the performance of Islamic funds and their conventional counterparts.

In the second part of our analysis, we have shown that there is a significant overlap between SRI principles and the objectives of *Shariah* or *Maqasid-al-Shariah.* Islamic funds, in general, are considered as Morally Responsible Investment (MRI) funds (Ghoul and Karam, 2007) and also *Halal* from a *Shariah* perspective. However, this does not always mean they have a direct contribution to the SDGs financing. Thus, to make Islamic funds effective tools for financing SDGs, *Shariah-compliant* investors need to consider adopting an ethico-legal *Shariah* compliance approach in the selection of their investments. Complying with legal principles while achieving economic objectives as well as social and environmental impacts is important to achieve the ultimate objective of Islamic law i.e. promoting well-being and preventing harm.

Therefore, the broader implication of this study is that synergies between Islamic funds and SRIs can play a significant role to fill, or at least to narrow, the existing SDGs financing gaps especially in Muslim countries in which SRIs funds are not widely contributing to SDGs financing. Accordingly, we suggest developing a new asset class that is both *Shariah*-compliant and incorporates SDGs and SRI principles. The objective is to promote the achievement of the SDGs and therefore create a positive impact on the society. This asset class will target both *Shariah*-compliant and impact investors and will apply both *Shariah* screening and SRI strategies in the investment screening process.

However, the role of various stakeholders including policymakers and *Shariah* scholars is critical to promote the development of the new asset class. In this regard, shariah scholars can promote the integration of SRI and ESG considerations into the *Shariah-*compliance review process of Islamic funds. Policymakers and regulatory bodies, on the other hand, can support the initiative by developing relevant policies and guidelines to promote transparency and standardization. The synergy will also have a positive implication on portfolio management. Since the new asset class (“*Shariah-compliant SRI*”) will have its own risk-return characteristics and investor base, it increases the diversification opportunities for portfolio managers.

Finally, we believe that fundamental research works should be conducted on the possibility of synergies between SRIs and Islamic finance in general. Future researches should further study the impact of synergies in the short and the long-term. The scope of the study should also go beyond achieving SDGs to integrate other development objectives.

## Declarations

### Author contribution statement

A.J. Yesuf: Conceived and designed the experiments; Performed the experiments; Analyzed and interpreted the data; Wrote the paper.

D. Aassouli: Conceived and designed the experiments; Analyzed and interpreted the data; Wrote the paper.

### Funding statement

This research did not receive any specific grant from funding agencies in the public, commercial, or not-for-profit sectors.

### Competing interest statement

The authors declare no conflict of interest.

### Additional information

No additional information is available for this paper.

## Footnotes

1 Majority of the world least developed (poor) countries are found in this organization. Poverty, hunger, lack of quality education and health care, gender inequality, clean water, and sanitation, etc. are the main problems in these countries. Of the 1.8 billion population of the OIC member countries, a total of 465 million people was identified as multidimensional poor, representing 26% of the total population of the OIC countries and 29% of the world's total multidimensional poor in 2014 (SESRIC, 2015).

2 *Riba is an increase, addition, unjust return, or advantage obtained by the lender as a condition of a loan. Any risk-free or “guaranteed” rate of return on a loan or investment is Riba. Riba in all its forms is prohibited in Islam. In conventional terms, Riba and “interest” are used interchangeably, although the legal notion extends beyond mere interest*.

3 *Gharar is one of the fundamental prohibitions in Islamic finance. It is a concept that covers certain types of haram uncertainty whereby one or more parties stand to be deceived through ignorance of an essential element in the contract. Gambling is a form of Gharar because the gambler is ignorant of the result of the gamble. The prohibition on Gharar is often used as the grounds for criticism of conventional financial practices such as short selling, speculation and derivatives*.

4 *Maysir is game of chance or gambling. It is one of the fundamental prohibitions in Islamic finance. The prohibition on Maysir is often used as grounds for criticism of conventional financial practices such as speculation, conventional insurance and derivatives*.

5 *Source: AAOIFI, Financial Accounting Standard No. 14, Investment Funds, Appendix**B*.

6 *According to the 2015 OIC report, a total of 465 million people in OIC member countries were considered as multidimensional poor, which accounted for 29% of the world total multidimensional poor in 2014* (SESRIC, 2015)*. At the end of 2013, out of the 57 OIC member countries only 8 countries achieved the MDG 1 (Eradicating extreme hunger and poverty) targets, 4 countries achieved MDG 4 (which was reducing Child Mortality) & MDG 5 (Improving maternal health), and 22 OIC member countries are on track to reach the MDG 6 (Fight HIV/AIDS, Malaria and other diseases). During the year, the majority of OIC member countries were also seriously lagging behind in achieving the MDG 7 (Ensure Environmental Sustainability) and only two countries early achieved this goal* (SESRIC, 2013)*. In 2015, twenty-three OIC member countries were under the low human development group. During the same year, the OIC's average Human Development Index value was 0.636 which was below the average HDI for developing countries (0.668) and the world average of 0.717* (UNDP, 2016)*. There are still a significant number of OIC member countries where children stay out of school. Despite putting education as one of our criteria to select Islamic funds, based on our sample, we were unable to find a fund that invests in education or education-related activities. We believe that the reason we could not find a single fund investing in education may be due to the unavailability of data on Islamic funds*.

7 *“Maqasid al-Shari'ah may be stated simply as the higher objectives of the rules of the Shari'ah, the observance of which, facilitate the normal functioning of society by enhancing the public good (maslaha), this implies avoiding actions likely to harm to individuals and society. The intent, objective and purpose is simply to achieve social and economic justice as well as enhancing the welfare of the community. Maqasid Al-Shariah classified by the scholars into five elements of preservations. The objective of Islamic rulings is mainly to protect these five elements from any harms. These preservations are known as: Protection of Faith or religion (din), Protection of Life (nafs), Protection of Lineage (nasl), Protection of Intellect (‘aql), and Protection of Property (mal). Maqasid al-Shari'ah is not restricted to just prohibitive rulings and discouraging immoral conduct and harmful actions; it also places value on promoting and encouraging righteous behavior, moral conduct and such acts of goodness that are for the public good. The maqasid reflects a holistic view of life emphasized in Islam and has to be looked at as a way of life, as a whole and not in parts.” (Institute of Islamic Banking and Finance)*.

8 *The responsibility of ethical Committee on Socially Responsible Investing is to advise the Finance and Investment Committee regarding ethical, social, and environmental issues that might influence the management of the funds.*

9 *In SRI, Best-in Class is an approach where the leading companies with regard to ESG criteria from each individual sector or industry group are identified and included in the portfolio*.

## References

[bib1] Abdul Rafay U.J.G., Muhammad A.I. (2017). Investigating the performance of Islamic mutual funds: evidence from an Emerging economy. City Univ. Res. J..

[bib2] Abdullah F., Hassan T., Mohamad S. (2007). Investigation of performance of Malaysian Islamic unit trust funds: comparison with conventional unit trust funds. Manag. Finance.

[bib3] Agussalim, Limakrisna Nandan, Ali Hapzi (2017). Mutual funds performance: conventional and Sharia product. Int. J. Econ. Financ. Issues.

[bib4] Anand P., Cowton C.J. (1993). The ethical investor: Exploring dimensions of investment Behavior. J. Econ. Psychol..

[bib5] Argandoña A., Sarsa D. (2000). Ethical Funds as a Tool for Promoting Ethics in Business. Technical Report.

[bib6] Bacon Carl R. (2008). Practical Portfolio Performance Measurement and Attribution.

[bib7] Beal D., Goyen M. (1998). Putting your money where your mouth is a profile of ethical investors. Financ. Serv. Rev..

[bib8] Bello Zakri Y. (2005). Socially responsible investing and portfolio diversification. J. Financ. Res..

[bib9] Boo Y.L., Li B., Shan Ee M., Rashid M., Shan M., Ling Y. (2016). Islamic or conventional mutual funds: who has the upper hand? Evidence from Malaysia. Pac. Basin Finance J..

[bib10] Brammer S.J., Pavelin S. (2006). Corporate reputation and social performance: the importance of fit. J. Manag. Stud..

[bib11] Brooks C. (2008). Introductory Econometrics for Finance.

[bib12] Brugnoni Alberto (2009). Smoke Signals. Islamic Banking Finance.

[bib13] Bruyn S.T. (1987). The Field of Social Investment.

[bib14] Camilieri M. (2017). Socially responsible and sustainable investing. Corporate Sustainability, Social Responsibility and Environmental Management.

[bib76] Carhart M.M. (1997). On persistence in mutual fund performance. J. Finance.

[bib15] Chatzitheodorou K., Skouloudis A., Evangelinos K., Nikolaou I. (2019). Exploring socially responsible investment perspectives: a literature mapping and an investor classification. Sustain. Prod. Consumpt..

[bib16] Chong J., Her M., Phillips G.M. (2006). To sin or not to sin? Now that’s the question. J. Asset Manag..

[bib17] Chowdhury M.A.F., Masih M. (2015). Socially Responsible Investment and Shariah Compliant Investment Compared: Can Investors Benefit from Diversification? an ARDL Approach.

[bib18] Climent F., Molla P., Soriano P. (2020). The investment performance of U.S. Islamic mutual funds. Sustainability.

[bib19] Cowton C. (1992). The ethics of advertising: do investors care?. Int. J. Advert..

[bib20] Elfakhani S., Hassan M.K., Sidani Y., Hassan M. Kabir, Lewis Mervyn K. (2007). Islamic mutual funds. Handbook of Islamic Banking.

[bib21] EUROSIF (2018). European SRI study. http://www.eurosif.org/wp-content/uploads/2018/11/European-SRI-2018-Study.pdf.

[bib22] Farid Abderrezak (2008). The Performance of Islamic Equity Funds: A Comparison to Conventional, Islamic and Ethical Benchmarks.

[bib75] Fama E.F., French K.R. (1993). Common risk factors in the returns on stocks and bonds. J. Financ. Econ..

[bib23] Frank K.R., Brown Keith C. (2012). Investment Analysis & Portfolio Management.

[bib24] GABV (Global Alliance for Banking on Values) (2017). Real Economy – Real Returns: the Business Case for Values-Based Banking.

[bib25] Gait Alsadek, Worthington Andrew (2008). An empirical survey of individual consumer, business firm and financial institution attitudes towards Islamic methods of finance. Int. J. Soc. Econ..

[bib26] Ghoul Wafica, Karam Paul (2007). MRI and SRI mutual funds: a comparison of Christian, Islamic (morally responsible investing), and socially responsible investing (SRI) mutual funds. J. Invest..

[bib27] Global Impact Investing Network (GIIN) (2018). Roadmap for the Future of Impact Investing: Reshaping Financial Markets. https://thegiin.org/assets/GIIN_Roadmap%20for%20the%20Future%20of%20Impact%20Investing.pdf.

[bib28] Global Sustainable Investment Alliance (GSIA) (2016). Global Sustainable Investing Review. http://www.gsi-alliance.org/trends-report-2018/.

[bib29] Global Sustainable Investment Alliance (GSIA) (2018). Global Sustainable Investing Review. http://www.gsi-alliance.org/trends-report-2018/.

[bib30] Haigh M. (2007). What counts in social managed investments: evidence from an international survey. Adv. Publ. Interest Account..

[bib31] Hayat Raphie, Kraeussl Roman (2011). Risk and return characteristics of Islamic equity funds. J. Emerg. Markets Rev..

[bib32] Heard J. (1978). Investor responsibility: an idea whose time has come?. J. Portfolio Manag..

[bib33] Hoepner Andreas GF., Rammal Hussain G., Rezec Michael (2011). Islamic mutual funds' financial performance and international investment style: evidence from 20 countries. Eur. J. Finance.

[bib34] Hussein K., Omran M. (2005). Ethical investment revisited: evidence from Dow Jones Islamic indexes. J. Invest..

[bib35] Islamic Research and Training Institute (IRTI) (2017). I for impact: blending Islamic finance and impact investing for the global goals. A Report Published by the United Nations Development Programme, Istanbul International Center for Private Sector in Development.

[bib36] Jensen Michael C. (1968). The performance of mutual funds in the period 1945–1964. J. Finance.

[bib37] Khatkhatay M.H., Nisar S. (2006). Sharīʿah compliant equity investments: an assessment of Current screening Norms. Proceedings of the Seventh Harvard University Forum on Islamic Finance.

[bib38] Kreander N., Gray R.H., Power D.M., Sinclair C.D. (2005). Evaluating the performance of ethical and non-ethical funds: a matched pair analysis. J. Bus. Finance Account..

[bib39] Lewis A., Mackenzie C. (2000). Support for investor activism among UK ethical investors. J. Bus. Ethics.

[bib40] Lowry R.P. (1993). Good Money: A Guide to Profitable Social Investing in the ’90s.

[bib41] Luc Renneboog, Ter Horst Jenke, Zhang Chendi (2008). Socially responsible investments: institutional aspects, performance, and investor behavior. J. Bank. Finance.

[bib42] Malkiel B.G., Quandt R.E. (1971). Moral issues in investment policy. Harv. Bus. Rev..

[bib43] Mallin C., Saadouni B., Briston R. (1995). The financial performance of ethical investment funds. J. Bus. Finance Account..

[bib77] McLeod W., van Vuuren G. (2004). Interpreting the Sharpe ratio when excess returns are negative. Invest. Anal. J..

[bib78] Miglietta F., Forte G. (2011). A comparison of socially responsible and islamic equity investments. J. Money Invest. Bank..

[bib44] Mansor F., Bhatti M.I. (2011). The Islamic mutual fund performance: new evidence on market timing and stock selectivity.

[bib45] Maroof Lubna (2020). Are Islamic mutual funds exposed to lower risk compared to their conventional counterparts? Empirical evidence from Pakistan. ISRA Int. J. Islamic Finance.

[bib46] McLachlan J., Gardner J. (2004). A comparison of socially responsible and conventional investors. J. Bus. Ethics.

[bib47] Merdad Hesham, Kabir Hassan M., Alhenawi Yasser (2010). Islamic versus conventional mutual funds performance in Saudi Arabia: a case study. JKAU: Islamic Econom..

[bib48] Niculescu Mara (2017). Impact investment to close the SDG funding gap. https://www.undp.org/content/undp/en/home/blog/2017/7/13/What-kind-of-blender-do-we-need-to-finance-the-SDGs-.html.

[bib49] O’Neil R.F., Pienta D.A. (1994). Economic criteria versus ethical criteria toward resolving a basic dilemma in business. J. Bus. Ethics.

[bib50] Reddy Krishna, Mirza Nawazish, Naqvi Bushra, Fu Mingli (2017). Comparative risk adjusted performance of Islamic, socially responsible and conventional funds: evidence from United Kingdom. Econ. Modell..

[bib51] Refinitiv (2019). Islamic Finance Development Indicator: Shifting Dynamics. https://icd-ps.org/uploads/files/IFDI%202019%20DEF%20digital1574605094_7214.pdf.

[bib52] Rosen B.N., Sandler D.M., Shani D. (1991). Social issues and socially responsible investment behavior: a preliminary empirical investigation. J. Consum. Aff..

[bib53] Rudd A. (1981). Social-responsibility and portfolio performance. Calif. Manag. Rev..

[bib54] Sauer D.A. (1997). The impact of social-responsibility screens on investment performance: evidence from the Domini 400 social index and Domini equity mutual fund. Rev. Financ. Econ..

[bib55] Schröder M. (2007). Is there a difference? The performance characteristics of SRI equity indices. J. Bus. Finance Account..

[bib56] SESRIC (2013). OIC strategic health programme of action 2013-2022 (OIC-SHPA). Statistical, Economic and Social Research and Training Centre for Islamic Countries (SESRIC), Ankara/Turkey.

[bib57] SESRIC (2015). The state of children in OIC member countries. Statistical, economic and social research and training Centre for Islamic Countries (SESRIC); Ankara/Turkey. http://www.sesric.org/files/article/506.pdf.

[bib58] Shah Zaidi M.A., Zulkefly A.K., Zurina K. (2004). The performance of Islamic unit trust funds in Malaysia: does persistence exists?. J. Muamalat Islamic Finance Res..

[bib59] Shamsher M., Annuar M., Taufiq H. (2000). Investment in unit trusts: performance of active and passive funds. Proceedings of FEP Seminar 2000: Issues in Accounting and Finance.

[bib60] Sharpe W.F. (1966). Mutual fund performance. J. Bus..

[bib61] SRI Service (2019). Different sustainable and responsible investment approaches. https://www.sriservices.co.uk/about-sri/green-and-ethical-investment-approaches.

[bib62] Stanley M., Jaffery S., Jaffer Sohail (2009). Can Islamic asset management aim a little higher? Achieving maturity and the impact of socially responsible investment. Islamic Wealth Management: A Catalyst for Global Change and Innovation.

[bib63] Statman M. (2006). Socially responsible indexes: composition, performance and tracking-error. J. Portfolio Manag..

[bib64] Taib F., Isa M. (2007). Malaysian unit trust aggregate performance. J. Manag. Finance.

[bib65] Thomson Reuters (2018). Islamic finance development report: building momentum. https://repository.salaamgateway.com/images/iep/galleries/documents/20181125124744259232831.pdf.

[bib66] Treynor J. (1965). How to rate management of investment funds. Harv. Bus. Rev..

[bib67] UNDP (2016). Organization of Islamic Cooperation (OIC): statistical briefing based on the 2016 human development report; UNDP human development report. http://hdr.undp.org/sites/default/files/briefing_stat_oic_full.pdf.

[bib69] UNEP (2017). UN environment Inquiry annual overview. http://unepinquiry.org/wp-content/uploads/2018/01/UN_Environment_Inquiry_Annual_Overview_2017.pdf.

[bib70] Van L. Luc, Reza Z.J., Lohr Albert, Veleva Milena V. (2010). Some critical thoughts on a possible synergy between SRI and Islamic finance. Finance and Ethics – Das Potential von Islamic Finance, SRI, Sparkassen.

[bib71] Weigand E.M., Brown K.R., Wilhem E.M. (1996).

[bib72] Yaacob M.H., Yakob N.A. (2002). portfolio formation using Islamic-approved stocks in Malaysia. Cap. Markets Rev..

[bib73] Yildirim Ramazan, Ilhan Bilal (2018). Shari’ah screening methodology- new Shari’ah compliant approach. J. Islamic Econom. Banking Finance.

[bib74] Zahari A.R., Shanmugam B., Jaffer Sohail (2009). Socially responsible investing. Islamic Wealth Management: A Catalyst for Global Change and Innovation.

